# *COASY* variant as a new genetic cause of riboflavin-responsive lipid storage myopathy

**DOI:** 10.1038/s41421-023-00641-0

**Published:** 2024-02-27

**Authors:** Yilei Zheng, Tongling Liufu, Bing Wen, Chao Zhou, Lingchun Liu, Yusen Qiu, Wenquan Zou, Wei Zhang, Yu Li, Jianfeng Pei, Yiheng Zeng, Wanjin Chen, Chunhua Zhang, Yun Yuan, Guochun Wang, Chuanzhu Yan, Xin Lu, Jianwen Deng, Zhaoxia Wang, Daojun Hong

**Affiliations:** 1 Department of Neurology, The First Affiliated Hospital, Jiangxi Medical College, Nanchang University, Nanchang, Jiangxi China; 2 Department of Neurology, Peking University First Hospital, Beijing, China; 3 Department of Neurology, Qilu Hospital of Shandong University, Jinan, Shandong China; 4 State Key Laboratory of Molecular Developmental Biology, Institute of Genetics and Developmental Biology, Chinese Academy of Sciences, Beijing, China; 5 Department of Neurology, The First People’s Hospital of Yunnan Province, Kunming, Yunnan China; 6 Intelligence Pharma, Beijing, China; 7 Center for Quantitative Biology, Academy for Advanced Interdisciplinary Studies, Peking University, Beijing, China; 8 Department of Neurology, First Affiliated Hospital of Fujian Medical University, Fuzhou, Fujian China; 9 Department of Research & Development of MILS International, Yokohama, Japan; 10 Department of Rheumatology, China-Japan Friendship Hospital, Beijing, China

**Keywords:** Mechanisms of disease, Gene expression profiling

Dear Editor,

Human coenzyme A synthase (*COASY*) encodes a bifunctional enzyme containing 4’PP adenyltransferase (PPAT) and dephospho-CoA kinase (DPCK) domains that catalyzes the last two steps of de novo CoA biosynthesis (Supplementary Fig. S1)^1^. Biallelic *COASY* variants have been associated with severe neurodegenerative diseases^2,3^. However, no muscular disorders associated with *COASY* have been reported until now. Here, we found that biallelic *COASY* variants can be a novel genetic cause of riboflavin-responsive lipid storage myopathy (RR-LSM). RR-LSM is a subtype of lipid metabolic disorders with onset age ranging from infancy to adulthood. It is characterized by muscle weakness or exercise intolerance sometimes triggered by precipitating factors, occasional extramuscular multi-system symptoms, excessive accumulation of lipid droplets (LDs) in myofibers, and dramatic responsiveness to riboflavin^4^. Although genetic causes of the majority of the disorders have been identified, those for late-onset RR-LSM remain unresolved.

**Fig. 1:**
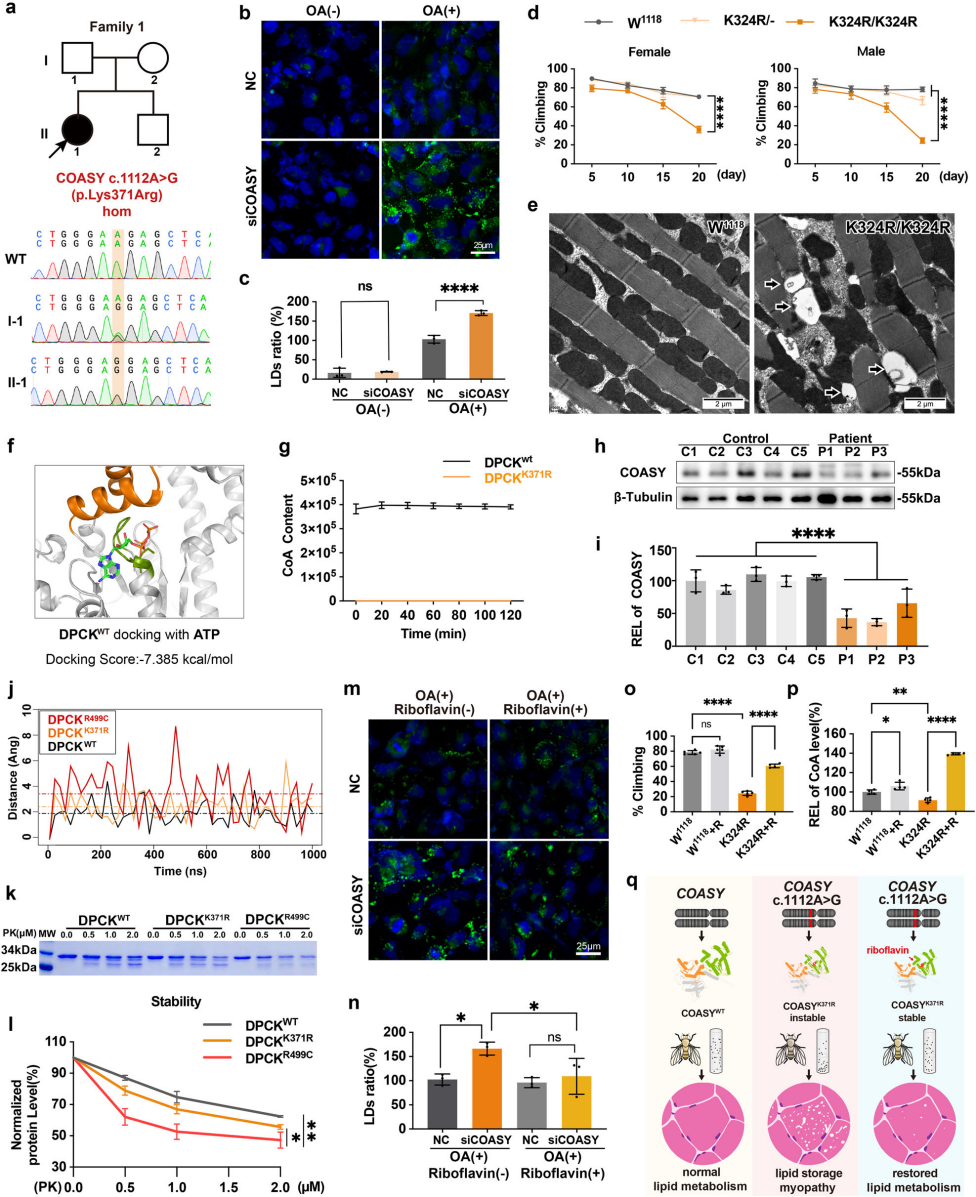
Biallelic *COASY* variant as a genetic cause of RR-LSM. **a** The index patient from the first family carried a homozygous missense variant c.1112A > G (p.Lys371Arg) in *COASY*. **b**, **c** BODIPY^TM^ staining showed no LD accumulation in normal control (NC) and *COASY*-knockdown HEK293T cells in normal culture medium, while numerous LDs accumulated in *COASY*-knockdown cells under OA overload (*n* = 3, ****P < 0.0001). **d** Female (left) and male (right) flies with homozygous p.Lys342Arg showed progressive locomotor deficits, as compared to heterozygous p.Lys342Arg or WT W^1118^ flies (*n* = 115–137, ****P < 0.0001). **e** Electron microscopy revealed obvious LDs accumulation in the muscles of homozygous p.Lys342Arg files at day 20 compared to WT W^1118^ flies. **f** MD analysis indicated that the P-loop (Green) containing p.Lys371 in the DPCK domain can bind with ATP. **g** Purified DPCK^K371R^ protein (orange) lost the ability to produce CoA in vitro compared to WT DPCK^WT^ (black). **h**, **i** Immunoblotting showed that the COASY levels in muscle biopsies of RR-LSM patients (P1–3) were significantly decreased by approximately half of those in age-matched controls (C1–5) (Control 100 ± 11.93 vs patient 48.43 ± 18.57, *n* = 3, ****P < 0.0001). **j** The 3 trajectories (DPCK^WT^, DPCK^K371R^ and DPCK^R499C^) indicated the distance of the αC atom at Arg503 compared to the one of initial position during the process of 1000 ns MD simulation. **k**, **l** DPCK^R499C^ was more susceptible to be incrementally degraded by proteinase K at different concentrations compared to DPCK^WT^ and DPCK^K371R^ in vitro (*n* = 3, *P < 0.05). **m**, **n** Riboflavin supplement significantly decreased the accumulation of LDs in *COASY-*knockdown cells under OA overload (*P < 0.05). **o** Riboflavin treatment significantly improved the locomotor of homozygous p.Lys342Arg files at day 20 (*n* = 139–170, ****P < 0.0001). **p** The CoA level in homozygous p.Lys342Arg flies was significantly lower than that of the WT W^1118^, but could be restored by riboflavin supplementation (*P* < 0.0001). **q** Schematic model of pathogenic mechanism in *COASY*-related RR-LSM.

To search for the genetic cause of these unsolved RR-LSM patients, we conducted whole-exome sequencing (WES) on 28 RR-LSM patients with unidentified causative genes from 294 patients diagnosed with RR-LSM based on clinical and pathological criteria at five neuromuscular disease centers. Among these patients, 266 people carried variants in the gene encoding electron transfer flavoprotein dehydrogenase. WES data analysis revealed that 16 out of 28 patients were identified to carry biallelic *COASY* variants (Fig. 1a). The clinical and myopathological features of these 16 patients were illustrated in Supplementary Figs. S2–S5 and Supplementary Tables S1, S2. No iron deposits were observed in the brains of 4 patients who underwent sensitivity-weighted imaging, with the oldest patient being 43 years old. Additionally, among the 7 patients who underwent metabolic screening, 4 patients showed a metabolic profile of multiple acyl-coenzyme A dehydrogenase deficiency (MADD)^5^. Out of the 16 patients, 13 patients were homozygous for c.1112A > G (p.Lys371Arg), and the other 3 were compound heterozygous, with c.1112A > G in one allele and c.805_806insC(p.Leu269Profs*11), c.1018_1019insA(p.Met340Asnfs*11), or c.383C > T(p.Pro128Leu) in the other allele, respectively. The examined individuals displayed family co-segregation (Supplementary Figs. S6, S7). Four variants were novel and predicted as deleterious by in silico analysis with highly evolutional conservation (Supplementary Fig. S8). The p.Lys371Arg, located in the DPCK domain, was a hotspot variant with an allele frequency of 90.6% (29/32). Homozygosity mapping in 12 patients with c.1112A > G homozygote showed a 0.64 Mb common region containing the variant, and further SNP analysis revealed a shared haplotype in the 0.64 Mb region, suggesting a founder effect in these patients (Supplementary Fig. S9). These results suggested that the *COASY* gene was implicated as a new causative gene for RR-LSM.

To demonstrate whether loss-of-function *COASY* variants played a causative role in lipid metabolism disorder, we established cell and *Drosophila melanogaster* models. Our studies revealed that, although there was no significant accumulation of LDs in the *COASY*-knockdown cells in the normal culture medium, the LDs significantly increased after oleic acid (OA) overload (Fig. 1b, c; Supplementary Fig. S10). The dysfunction of lipid metabolism resulting from lipid overload stress was highly consistent with the clinical phenotype of *COASY*-related RR-LSM patients, particularly case 2, who developed muscle weakness after a high-fat diet (Supplementary Table S1). Compared to wild-type (WT) *Drosophila*, *Drosophila* homozygous for the p.Lys342Arg in Ppat-Dpck (equivalent to p.Lys371Arg in human COASY) generated by CRISPR/Cas9, exhibited substantial LD accumulation in muscle fibers and showed a significant decrease in locomotor ability (Fig. 1d, e; Supplementary Fig. S11). Neurodegeneration is usually characterized by vacuole formation in the *Drosophila* brain, and thus we carefully examined the brain sections. Brains from p.Lys342Arg flies displayed no detectable neurodegenerative vacuoles compared to WT flies, suggesting that the decrease in locomotor activity was associated with defects in the muscular system (Supplementary Fig. S12). Collectively, the LSM phenotype recapitulated in the cellular and *Drosophila* models suggested that *COASY* variants identified in patients were disease drivers.

Meanwhile, to investigate the impact of *COASY* deficiency on CoA biosynthesis, we selected the hotspot variant, p.Lys371Arg, for functional experiments. Molecular docking (MD) analysis indicated that p.Lys371 is located in the P-loop motif (365–372: GISGSGKS) of enzymatic active pocket in the DPCK domain, which forms the binding site for ATP, suggesting that the p.Lys371Arg variant could directly affect ATP hydrolysis in DPCK (Fig. 1f; Supplementary Fig. S13). HPLC analysis revealed that purified human DPCK^K371R^ was unable to convert dephospho-CoA to CoA in vitro, suggesting that CoA biosynthesis dependent on COASY pathway might be disrupted by the p.Lys371Arg variant (Fig. 1g; Supplementary Fig. S14). Consistently, the level of CoA in the p.Lys342Arg knock-in flies was significantly lower than that in the WT flies (Fig. 1p). These data suggested that *COASY* variants related to RR-LSM caused insufficient DPCK enzyme activity, leading to an impairment in CoA biosynthesis.

CoA, primarily synthesized intracellularly through the COASY protein, plays an important role as an acyl-group carrier and carbonyl activator in fatty acid metabolism^6^. Previous studies have shown that a complete loss of COASY was linked to lethal perinatal-onset pontocerebellar hypoplasia type 12^3^, while 5% of COASY preservation was associated with early-onset COASY protein-associated neurodegeneration (CoPAN)^2^ (Supplementary Table S3). Immunoblotting on biopsied muscles from *COASY*-related RR-LSM patients with homozygous p.Lys371Arg variant showed a significant reduction in COASY protein levels by approximately half of those in age-matched controls (Fig. 1h, i). The results suggested that the level of residual mutant COASY proteins might be associated with the heterogeneous phenotype of *COASY*-related disorders.

Subsequently, to investigate the mechanisms underlying the different levels of residual COASY proteins caused by different variants, we conducted protein stability studies on the p.Lys371Arg variant and the CoPAN-associated variant p.Arg499Cys as a positive control^7^. Under the ff14SB force field simulated by molecular dynamics, the trajectories of the αC atom at residue Arg503 in the catalytic lid domain (LID) of the three systems (DPCK^WT^, DPCK^K371R^ and DPCK^R499C^) indicated that variants could influence the flexibility of the LID region and expand the catalytic pocket, ultimately leading to increased instability of the mutant protein (Fig. 1j). Consistently, in vitro experiments of limited digestion by proteinase K demonstrated that the protein level of p.Lys371Arg DPCK exhibited only a slight decrease compared to the WT, while DPCK with p.Arg499Cys was much more susceptible to proteinase K digestion compared to the WT or p.Lys371Arg variant (Fig. 1k, l). These findings suggested that different *COASY* variants exerted varying impacts on protein stability, consequently contributing to divergent clinical manifestations. Notably, COASY protein also exhibited several other biological functions for mitotic fidelity and DNA damage repair in tumor cells^8,9^. Furthermore, it was reasonably proposed that in CoPAN patients, the severity of central nervous system symptoms might be profound, potentially overshadowing muscle-related symptoms and associated pathological changes^2,3^. Collectively, the phenotypes of *COASY*-related diseases might not solely hinge on residual enzyme activity but also be contingent upon the level of residual COASY protein.

Due to the therapeutic effectiveness of riboflavin for *COASY*-related LSM, we further examined the mechanism of flavin adenine dinucleotide (FAD) on mutant COASY protein. COASY protein comprises a Rossmann-like fold domain, in which an αβα fold structure can bind to the FAD molecule^10^. MD scores indicated that FAD could effectively bind to the catalysis pocket of p.Lys371Arg viriant, while the p.Arg499Cys located in the LID region disrupted the conformation of the substrate-binding pocket and blocked the binding of FAD (Supplementary Fig. S15). Consistently, the FAD precursor riboflavin significantly increased the level of COASY proteins in p.Lys371Arg expressing cells, but no detectable effects in p.Arg499Cys expressing cells (Supplementary Fig. S16). Furthermore, riboflavin treatment resulted in a significant reduction in the accumulation of LDs in *COASY*-knockdown cells under OA overload (Fig. 1m, n). In *Ppat-Dpck*^K324R^ knock-in flies, supplementation with 50 μM riboflavin resulted in a substantial reduction in the accumulation of LDs in muscle, a significant increase in CoA levels, and a marked improvement in motility performance (Fig. 1o, p; Supplementary Fig. S17). These findings suggested that FAD attenuated the clinical phenotype of *COASY*-related RR-LSM by stabilizing the p.Lys371Arg COASY protein (Fig. 1q). Additionally, considered that multiple flavoproteins play fundamental roles in cellular energy metabolism, riboflavin supplementation has the potential to enhance the conformation of variable flavoproteins and improve their stability. Therefore, longer follow-up and more functional assays are required for the efficacy of riboflavin in these patients.

In summary, we identified biallelic variants in *COASY* as a new genetic cause in 16 out of 28 genetically unresolved RR-LSM patients. By modeling the loss-of-function variant in cells and *Drosophila*, we have demonstrated that COASY defects lead to LSM. Importantly, our findings showed that riboflavin could stabilize the mutant COASY protein, offering therapeutic benefits for *COASY*-related RR-LSM, as well as a potential intervention for *COASY*-related neurodegenerative diseases.

### Supplementary information

COASY variant as a new genetic cause of riboflavin-responsive lipid storage myopathy
