# Supplementary material for: COASY variant as a new genetic cause of riboflavin-responsive lipid storage myopathy
Source: Cell Discov. 2024 Feb 27;10:25. doi: 10.1038/s41421-023-00641-0 (PMC10899607; doi:10.1038/s41421-023-00641-0)
Supplement: Supplementary file 1 — COASY variant as a new genetic cause of riboflavin-responsive lipid storage myopathy [file 41421_2023_641_MOESM1_ESM.pdf]

Supplemental information

COASY variant as a new genetic cause of riboflavin-responsive lipid storage myopathy

Yilei Zheng[1,2], Tongling Liufu[2], Bing Wen[3], Chao Zhou[4], Lingchun Liu[5], Yusen Qiu[1], Wenquan Zou[1], Wei Zhang[2], Yu Li[6], Jianfeng Pei[7], Yiheng Zeng[8], Wanjin Chen[8], Chunhua Zhang[9], Yun Yuan[2], Guochun Wang[10], Chuanzhu Yan[3], Xin Lu[10*], Jianwen Deng[2*], Zhaoxia Wang[2*], and Daojun Hong[1*]

***Correspondence:** Xin Lu (luxin_n@163.com) or Jianwen Deng (jianwendeng@pkufh.com) or Zhaoxia Wang (drwangzx@163.com) or Daojun Hong (hongdaojun@hotmail.com)

[1]Department of Neurology, The First Affiliated Hospital, Jiangxi Medical College, Nanchang University, China

[2]Department of Neurology, Peking University First Hospital, Beijing, China

Full list of author information is available at the end of the article

These authors contributed equally: Yilei Zheng, Tongling Liufu, Bing Wen, Chao Zhou, Lingchun Liu

Methods and Materials

Standard Protocol Approvals, Registrations, and Patient Consents

All clinical data and tissue samples were obtained after a written consent form was signed by each individual in compliance with the bioethics laws of China as well as the Declaration of Helsinki. This study was approved by the Ethics Committee of Peking University First Hospital (2019–181) and First Affiliated Hospital of Nanchang University ((2022)CDYFYYLK(06-028)).

Participants

A total of 91 independent cases with RR-LSM were initially recruited for this study between Jan 2011 and Dec 2022. Of them, 52 cases were recruited from the First Affiliated Hospital of Nanchang University (NCUFH), Nanchang, and 39 cases were recruited from the Peking University First Hospital (PKUFH), Beijing. In order to further expand the sample size of RR-LSM, an additional 203 unrelated index individuals with a clinicopathological diagnosis of RR-LSM were also recruited from other three centers including Qilu Hospital of Shandong University, Jinan, the First People's Hospital of Yunnan Province, Kunming, and China-Japan Friendship Hospital, Beijing. The diagnostic criteria included (1) myopathic fatigue or weakness, predominantly in the proximal limbs; (2) muscle pathological findings indicated LSM characterized by numerous lipid droplets accumulating in the muscle fibers on oil red O (ORO) stain; (3) clinical symptoms significantly responsive to riboflavin treatment in one month; (4) secondary muscle lipid metabolic disorders due to various reasons were excluded by a battery of laboratory examinations. Five normal muscle samples were obtained from adult participants who underwent surgery for the fracture.

Muscle collection and pathological examination

All patients were subjected to muscle biopsies as described previously[1]. Serial sections of the

frozen muscle in 8 μm were stained according to standard procedures with stains of histochemistry and enzyme histochemistry. A small part of the skeletal muscle and *Drosophila* thoracic muscles were processed by standard procedures and examined under an electron microscope.

Genetic screening

Genomic DNA was extracted from peripheral blood samples. Whole exome sequencing (WES) was commercially supported by Running Gene Inc. (Beijing, China). Synonymous, available intronic variants, and copy number variation (CNV) in the electron transfer flavoprotein dehydrogenase (*ETFDH*) gene, alpha ETF (*ETFA*), beta ETF (*ETFB*), flavin adenine dinucleotide synthetase 1 (*FLAD1*), and solute carrier family 25 member 32 (*SLC25A32*) genes were analyzed to exclude the potential variants that might be responsible for these genetically unsolved LSM patients[2-4]. Sanger sequencing was conducted to confirm the *COASY* variant in all affected members and available family members. The reference sequence of *COASY* was RefSeq NM_025233.6.

Due to the high incidence of c.1112A>G in these patients, the available variant call format (VCF) files of WES from 12 cases were initially analyzed by homozygosity mapping using the AutoMap tool to evaluate the founder effect[5]. SNP profiles for 12 cases were extracted from WES data using LINKDATAGEN[6]. MERLIN was used to estimate the haplotypes[7].

Molecular docking and molecular dynamic simulations

Molecular dockings were performed by Glide and the parameters of related docking were set as default. GROMACS was used to run the molecular dynamics (MD) simulations for the three systems with the ff14SB force filed was selected to descript the protein[8]. The COASY protein 3D structure (Q13057, AlphaFold) was rendered using PyMOL[9].

Plasmid construct

The cDNA encoding hDPCK domains (c.1338A-c.2148A) and full-length COASY (c.454A-c.2145C) fused with 3 × FLAG was synthesized and cloned into pET30-a and pcDNA3.1(+) expression vector, respectively. The c.1112A>G (p.Lys371Arg) was introduced using a site-directed mutagenesis kit. The c.1495C>T (p.Arg499Cys) variant was served as positive control associated with CoPAN[10].

Purification of recombinant hDPCK and in vitro analysis of hDPCK activity

The expression and purification of recombinant hDPCK in bacteria were carried out following previously established protocols[10]. The plasmid pET30-a (Novagen) containing wild-type or mutant human dephospho-CoA kinase (hDPCK) domain was transformed into E. coli BL21 (DE3). After overnight induction at 16°C, the bacteria were harvested and lysed. The lytic supernatant was loaded on a Ni-NTA beads (QIAGEN) column. Bound proteins were eluted with an imidazole gradient and analyzed by 10% SDS-PAGE followed by Coomassie Blue staining. After the correct molecular-weight proteins were analyzed by SDS-PAGE, the fraction containing His-hDPCK was concentrated, filtered, and loaded onto a HiPrep 16/60 Sephacryl S-300 HR (GE Healthcare). The proteins were digested with sumo protease, and hDPCK was collected by Ni column, filtered, and concentrated (1 mg/ml).

The recombinant wild-type or mutant hDPCK proteins were quantified by HPLC. The ultraviolet (UV) detector was 259 nm. Equal amounts of CoA and dephospho-CoA were precisely weighed, and prepared a standard solution with a concentration of 5 mM. The standard solution gradient was diluted into a reference solution with a final concentration of (100 μM, 20 μM, 10 μM, 5 μM, 0 μM). 2 μg purified protein samples after enzyme digestion were mixed with 1 mL of 50 mM Tris-

HCl (pH 8.0), 5 mM $MgCl_2$, 1 mM ATP, 0.1 mM dephospho-CoA buffer, and 10 μL samples were added into the liquid chromatography. A total of 7 time points (0 min, 20 min, 40 min, 60 min, 80 min, 100 min, 120 min) were recorded. These CoA compounds were quantified by peak area compared with the standards.

Cell culture, transfection, and RNA interference

HEK293T cells were maintained at Dulbecco's modified Eagle medium (DMEM; Gibco) supplemented with 10% fetal bovine serum (FBS) (Gibco), 0.06 mg/ml penicillin, and 0.1 mg/ml streptomycin (BI). Cells were cultivated in tissue culture plates (CORNING) at 37°C in an incubator with an atmosphere of 5% CO2 and 95% air. The expression of COASY in HEK293T cells was silenced by siRNA transfection for 24 h. To mimic metabolic stress, sodium oleate (OA) (250 μM, Sigma) was simultaneously added to the culture medium for 24 h. Riboflavin (530 μM, Sigma) for rescue was simultaneously added to the culture medium. After transient transfection of HEK293T cells with plasmids containing full-length *COASY* or variants for 12 hours and rescued by complete medium with or without 530 μM riboflavin (Sigma) for 24 h, cells were analyzed by Western blot. siRNA and plasmids were transfected into cells using Lipofectamine 3000 according to the manufacturer's instructions (Invitrogen). The sequences of siRNA oligonucleotides (JTSbio, CN) targeting human COASY were as follows: COASY-siRNA: 5'-CCUCUUGCAGAAGCGCAUUTT-3'.

Analysis of lipid droplets

HEK293T cells with *COASY*-knockdown were cultured on glass coverslips with/without OA overload or were rescued by riboflavin. The coverslips were washed three times with phosphate buffer saline (PBS) and stained with 2 μM BODIPY^{TM} 493/503 (Invitrogen) for 20min at 37°C. The coverslips were washed three times with PBS and then fixed in 4% (w/v) paraformaldehyde at

room temperature for 30min. The samples were mounted using ProLong^{TM} Gold Antifade mounting medium with DAPI (Invitrogen). Images were acquired by an inverted microscope ECLIPSE Ti2-E (Nikon).

Western blot analysis

Skeletal muscle tissues from *COASY* related RR-LSM cases, control subjects, and cultured cells were lysed with RIPA buffer. The lysates were analyzed according to the standard procedure of Western blot (WB) with specific antibodies, including anti-COASY (Abcam, ab129012), FLAG (Sigma, SAB4200071), β-tubulin (Proteintech, 10068-1-AP), and β-actin (Proteintech, 20536-1-AP). The intensities of the bands were quantified using ImageJ software.

Limited proteolysis analysis

Limited proteolysis experiments were performed on the above purified recombinant human $DPCK^{WT}$, $DPCK^{K371R}$ and $DPCK^{R499C}$. Proteolysis was initiated by adding proteinase K at different concentrations (0.5 μM, 1.0 μM, and 2.0 μM)[11]. Reactions were quenched by 100 μM of PMSF after 10 min. Subsequently, the samples were analyzed using SDS-PAGE and stained with Coomassie Blue. Control samples without proteinase K treatment were included for comparison.

***Drosophila* Models**

Using the CRISPR/Cas9 editing method, we generated the p.Lys324Arg variant in the *Drosophila Ppat-Dpck* gene, which corresponds to the p.Lys371Arg variant in the human *COASY* gene. Briefly, Cas9 mRNA was cloned into Plasmid MLM3613 (Addgene) vector. The plasmid and gRNA were mixed with DEPC water and injected into fly embryos. The genomic DNA of knock-in flies was extracted and the point mutation was validated. The F2 flies from positive F1 tubes were balanced with TM6B. All flies were reared at 25°C and 60% humidity with a 12-hour on/off light

cycle on standard cornmeal agar medium. Sex-specific climbing assays were performed using vials containing 20-30 flies[12].

Climbing assay

All flies were separated within 1 day after eclosion and transferred to new vials every 4 days throughout the assay period. For climbing assay, flies were lightly tapped down to the bottom of the vial, then were recorded the number of flies climbing above 5 cm within 15 seconds. Each climbing trial was repeated five times. The climbing assay was recorded every 5 days. In the riboflavin supply experiments, one-day-old flies were transferred to standard fly food supplementing with 50 μM riboflavin, which was freshly prepared before each experiment and provided to the flies.

Measurement of CoA levels in *Drosophila*

Prepare Drosophila samples and measure CoA levels according to the instructions provided by the manufacturer of the Coenzyme A Assay Kit (Sigma). In brief, 100 alive flies (approximately 85 mg) were transferred into a glass-Teflon Dounce homogenizer filled with 200 μL of ice-cold PBS and homogenized on ice for 20 strokes[12]. The homogenate was then transferred to a 1.5 mL 10 kDa Molecular Weight Cut-Off spin filter for centrifugation at 12000 × g for 10 min at 4 °C. Add between 25 μL deproteinized samples into duplicate wells of a black 96-well flat-bottom plate and bring to a final volume of 40 μL with Coenzyme A Assay Buffer. CoA concentration is determined by fluorometric (λEx = 535 nm/λEm = 587 nm). Samples tested for CoA levels were normalized to the corresponding protein concentrations by the BCA protein assay kit.

Statistical analysis

Statistical analyses were performed using GraphPad Prism version 9.0 (GraphPad Software).

Differences between the two groups were analyzed using an unpaired Student’s t-test. Ordinary one-way ANOVA was used to compare different patients and groups. The bar graphs with error bars represent mean ± standard deviation (SD) or median (Interquartile range, IQR), as appropriate.

Data availability

The original data that support the findings are available from the corresponding author (Daojun Hong) on reasonable request.

Supplementary figures

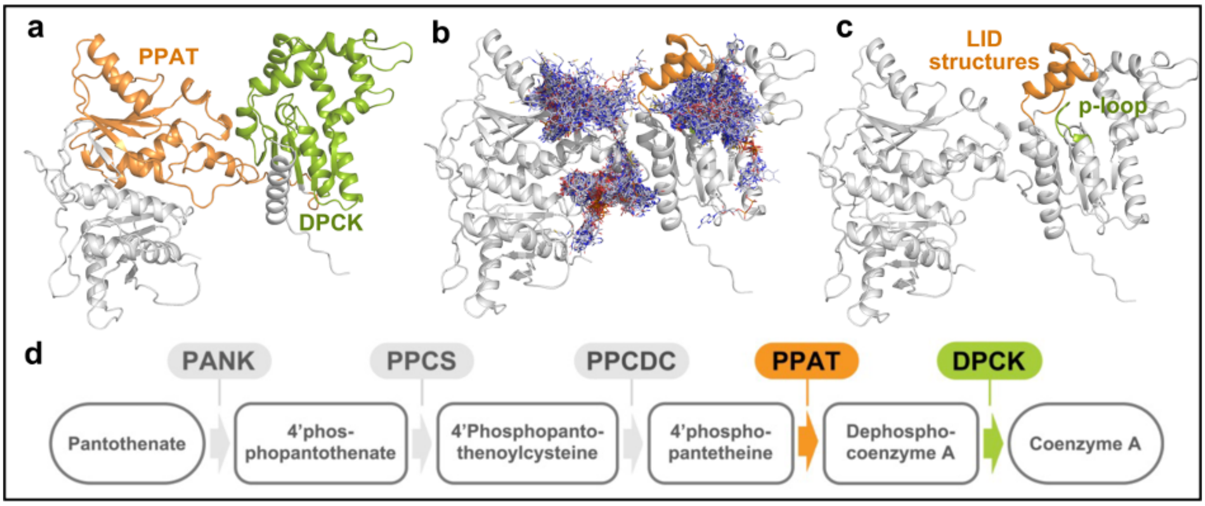

Fig. S1 Structure of COASY protein and schematic representation of CoA biosynthesis. a. The predicted 3D structure of COASY protein. 4'PP adenyltransferase (PPAT) domain (orange), dephospho-CoA kinase (DPCK) domain (green). **b** The predicted enzyme activity pockets on COASY proteins. **c** The location of the LID structuresand P-loop motif (365-372: GISGSGKS) in the structure of the COASY protein. **d** The synthetic pathway of intracellular CoA. PPAT and DPCK domains in COASY protein that catalyzes the last two steps of *de novo* CoA biosynthesis.

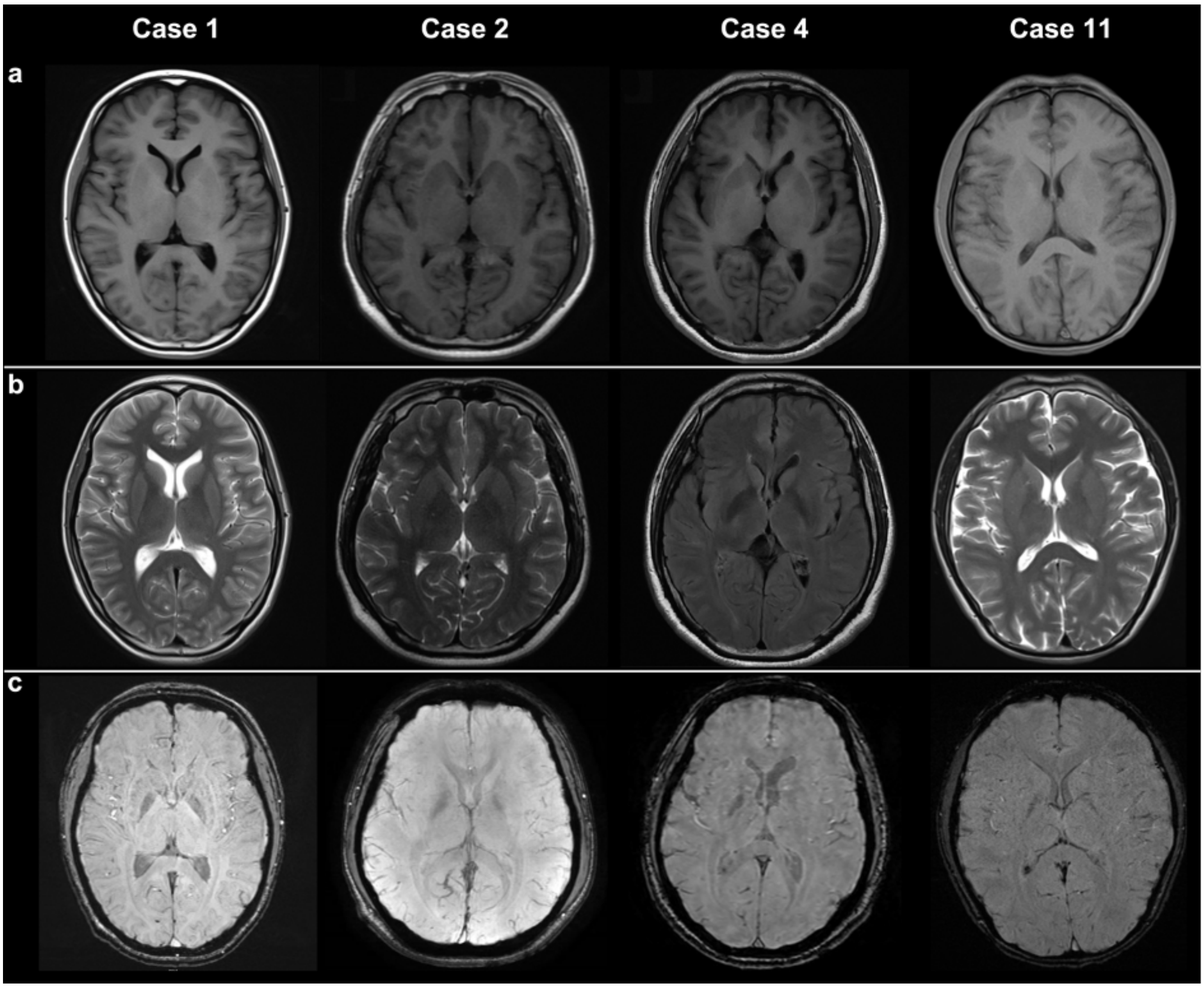

Fig. S2 Brain radiological features in *COASY*-related RR-LSM. a-c Brain MRI was normal on T1-weighted image (a), T2-weighted image (b), and susceptibility-weighted images (SWI) (c). No iron deposits were observed in the brain of the four patients who underwent SWI (case 1, case 2, case 4, and case 11, respectively).

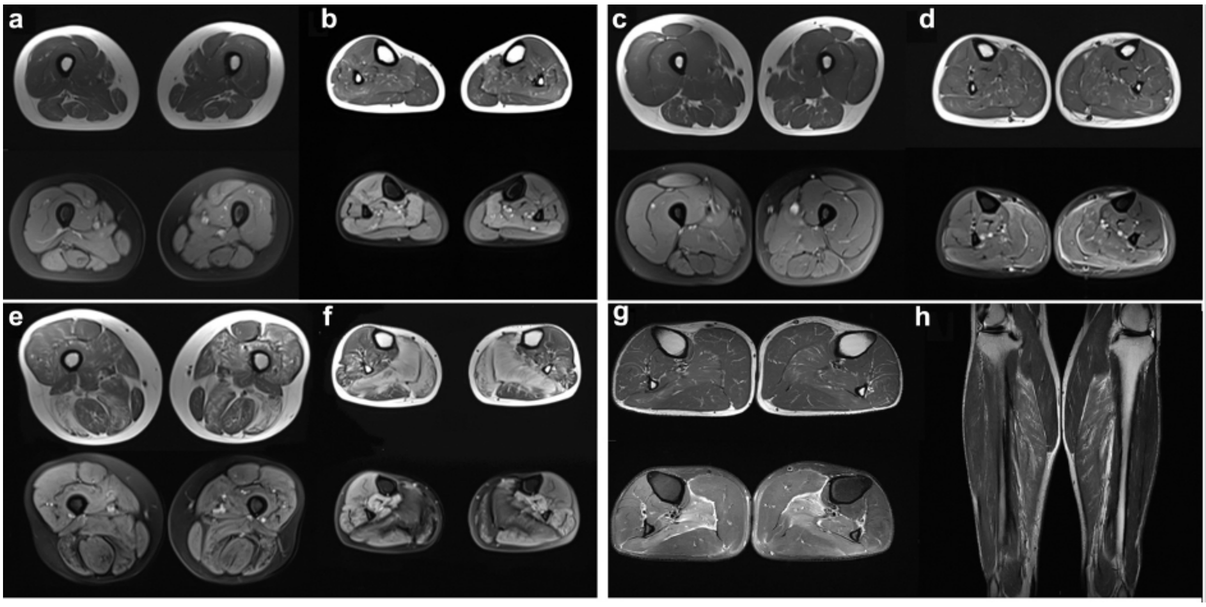

Fig. S3 Muscle radiological features in *COASY*-related RR-LSM. **a-b** Muscle MRI of case 1 showed slight fat infiltration and edema-like changes in biceps femoris longus (BFL) and semimembranosus (SM) muscles on thigh level and soleus (SO) muscle on leg level. **c-d** No obvious abnormalities were observed in case 2 on thigh level, while mild fatty infiltration and edema-like changes in SO muscle were observed, with posterior myofascial edema on leg level. **e-f** Muscle MRI of case 3 showed fat infiltration in quadriceps femoris, BFL, and SM muscles without edema-like change and a severe fat infiltration and edema-like change in the SO and medial gastrocnemius muscles on leg level. **g-h** Leg muscle MRI of case 4 showed mild fat infiltration and severe edema-like change in SO muscle with posterior myofascial edema.

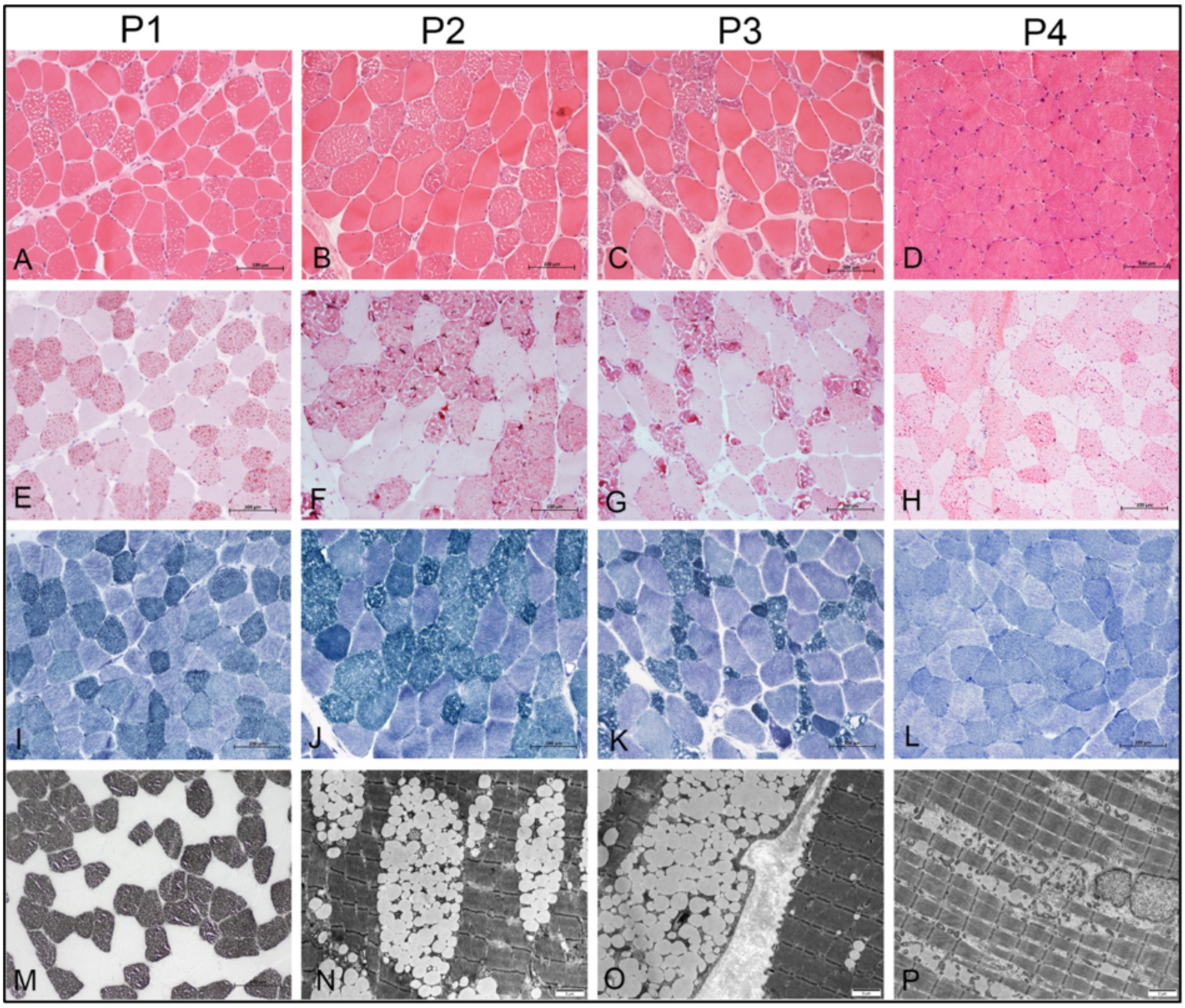

Fig. S4 Myopathological changes in *COASY*-related RR-LSM. a-d Many myofibers were filled with small round or irregular vacuoles on H&E staining in the in the representative case 1-4, respectively. **e-l** These vacuolar fibers were filled with lipid droplets on ORO staining (**e-h**) and exhibited numerous dark granules on NADH staining (**i-l**). **m** Lipid droplets predominantly accumulated in the type I fibers on ATPase staining with pH 4.3. **n-p** Ultrastructural examination revealed many lipid droplets deposited between myofibrils in cases 2-4, respectively.

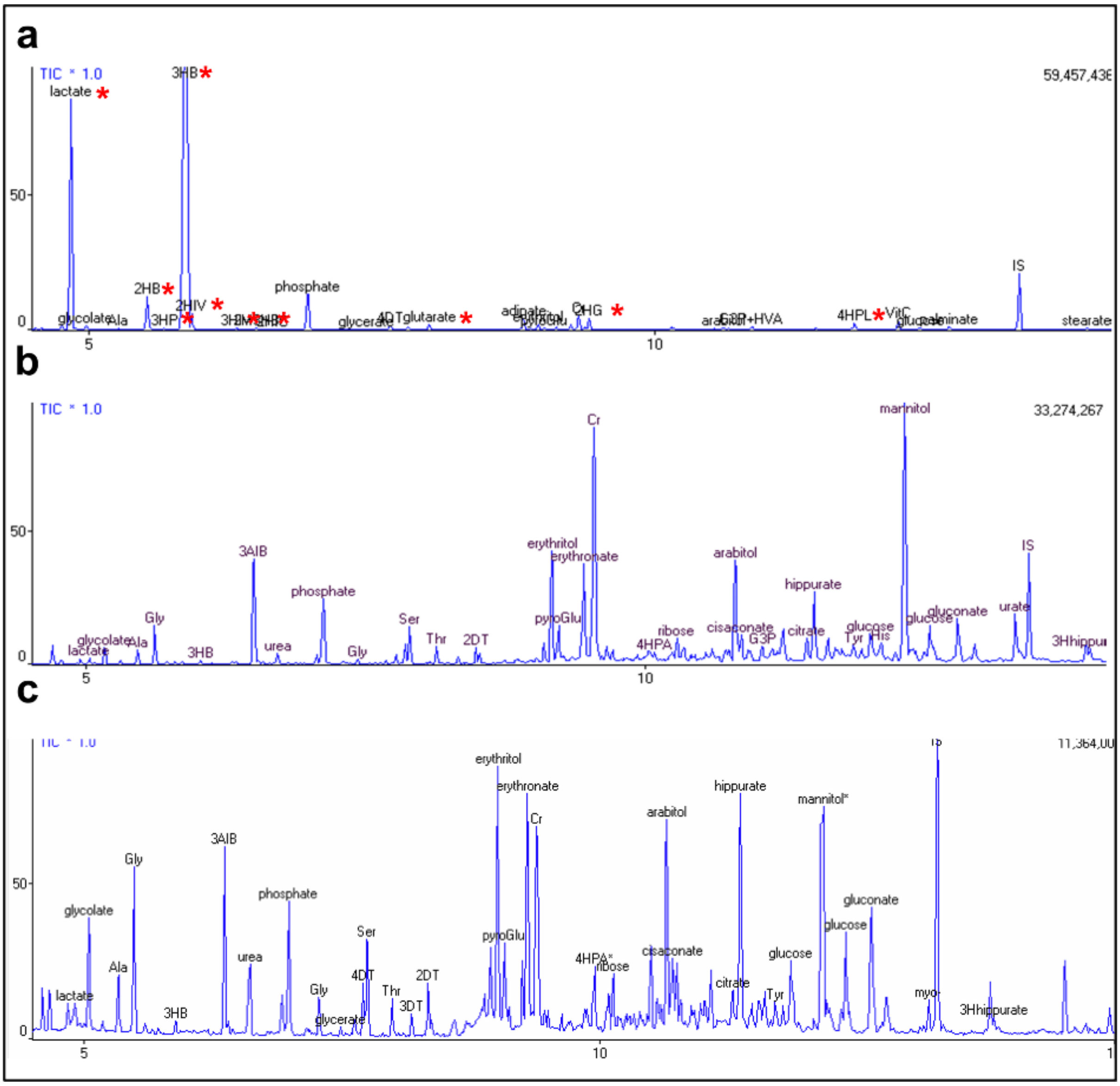

Fig. S5 Metabolic profiles of case 7 before and after riboflavin treatment. a The urine organic acid profiles of the patient on the day of acute onset showed abnormal increases in lactate, 2-hydroxybutyrate (2HB), 3-hydroxybutyrate (3HB), 2-hydroxyhexanoic acid (2HIV), 2-methyl-3-hydroxybutyrate (2M3HB), 2-hydroxyhexanoic acid (2HIC), glutarate, adipate, 2-hydroxyglutaric acid (2HG) and 4-hydroxyphenylactic acid (4HPL). **b** The abnormal profiles of urine organic acids in case 7 completely returned to normal after one month of riboflavin treatmen.**c** After a follow-up examination 10 months later, no abnormal metabolic products observed during the initial check

were found, and no other abnormalities were detected. All urinary metabolites during the remission period were normal.

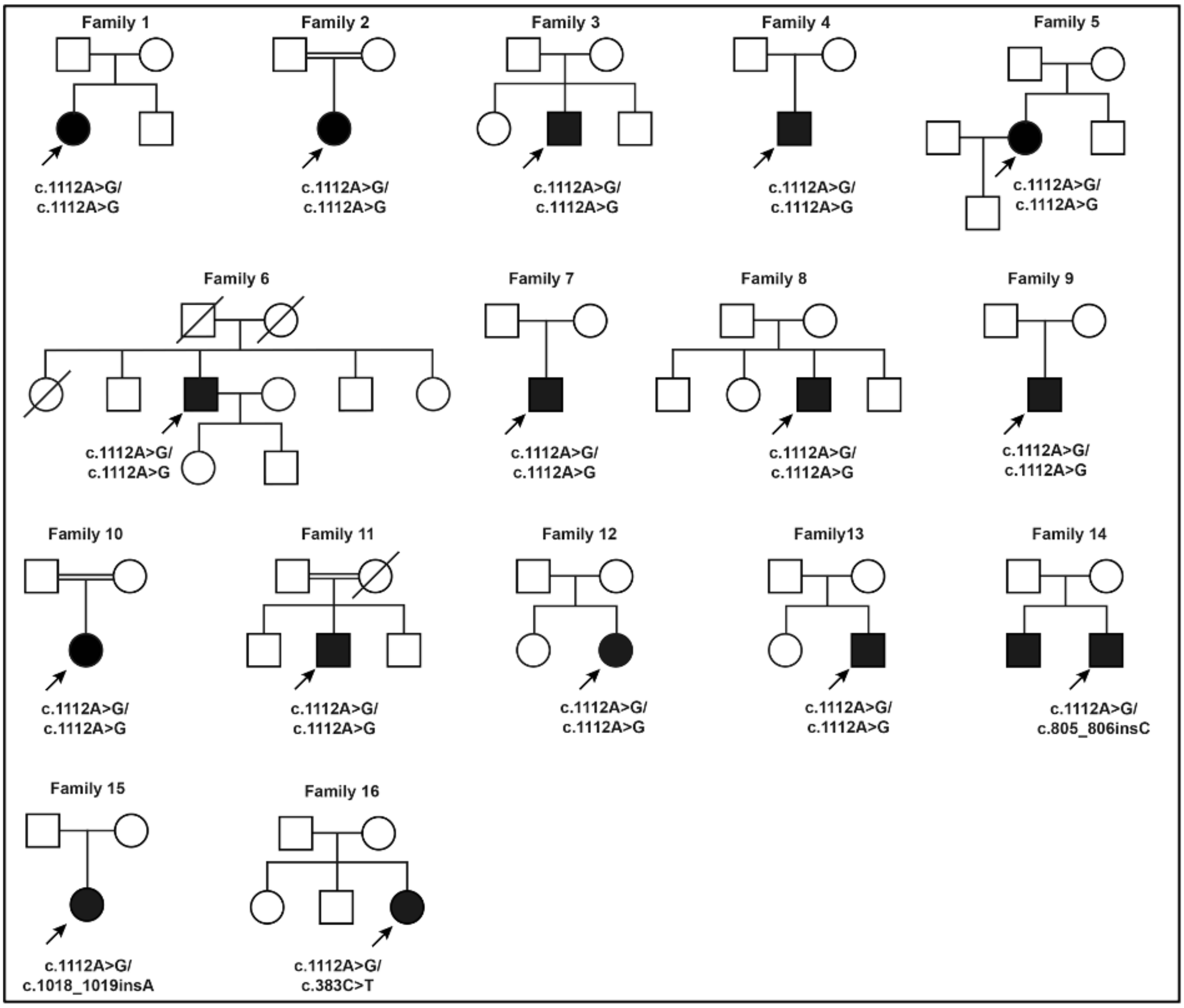

Fig. S6 Pedigrees of families carrying biallelic variants in *COASY*. The squares indicate males, the circles females, and the diagonal lines deceased individuals. Patients are indicated with filled shapes. Genotypes are provided in the index patients.

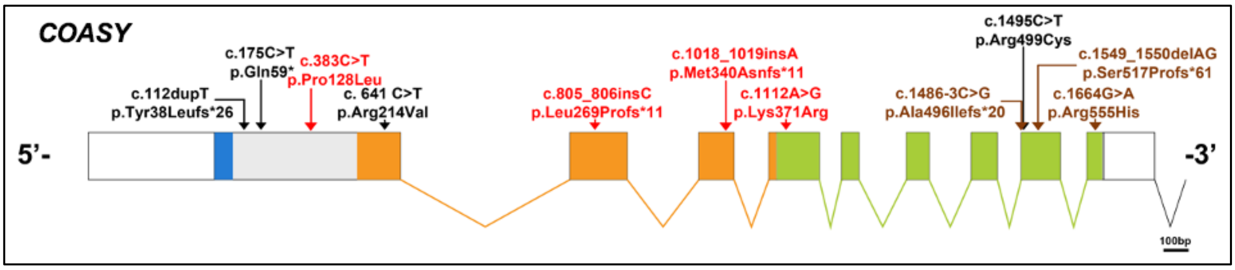

Fig. S7 Schematic representations of variants in *COASY*. The distribution of reported variants: the variants indicated by black fonts were associated with COASY protein-associated neurodegeneration (CoPAN), the variants indicated by brown fonts were associated with pontocerebellar hypoplasia type 12 (PCH12), the variants indicated by red font were associated with *COASY*-related RR-LSM (RefSeq.ENST00000393818.3). Region PPAT (Orange), Region DPCK (Green).

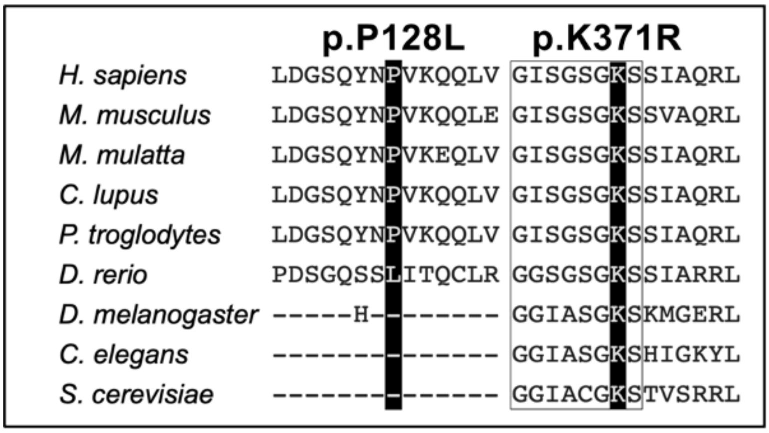

Fig. S8 Evolutional analysis of novel missense variants associated with *COASY*-related RR-LSM. The residue p.Pro128 was highly conserved in mammals. The residue p.Lys371 showed high conservation across species.

a

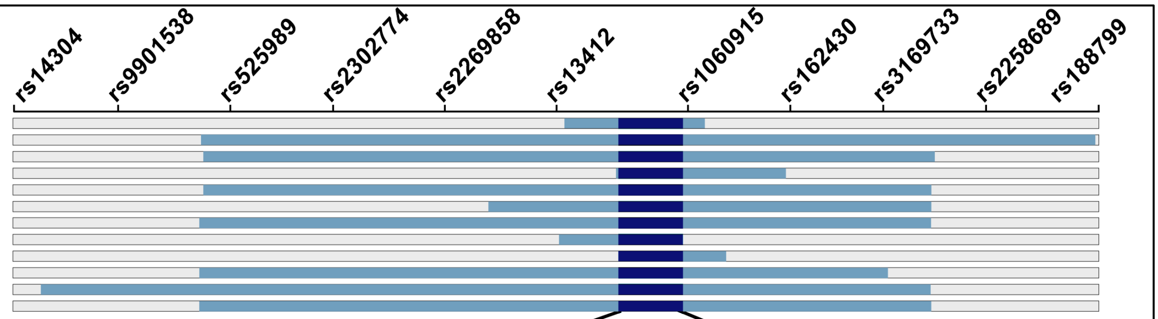

b

	rs14304	rs9901538	rs525989	rs2302774	rs2269858	rs13412	rs2074156	c.1112A>G	rs1553468	rs7214297	rs2229611	rs1060915	rs162430	rs1558085	rs1230095	rs188799
Case1	C/C	T/T	G/G	G/G	G/G	C/T	G/G	G/G	G/G	C/C	C/C	A/A	A/G	A/A	C/G	C/C
Case2	T/T	T/T	G/G	G/G	G/G	T/T	G/G	G/G	G/G	C/C	C/C	A/A	A/A	A/A	G/G	C/C
Case3	T/T	T/T	A/A	G/G	G/G	T/T	G/G	G/G	G/G	C/C	C/C	A/A	G/G	G/G	C/C	C/C
Case4	C/T	T/T	A/G	G/G	G/G	T/T	G/G	G/G	G/G	C/C	C/C	A/A	A/A	A/A	C/G	C/C
Case5	C/C	T/T	G/G	T/T	A/A	T/T	G/G	G/G	G/G	C/C	C/C	A/A	A/A	1/1	G/G	C/C
Case6	C/C	T/T	G/G	G/T	G/G	T/T	G/G	G/G	G/G	C/C	C/C	A/A	A/A	G/G	G/G	C/C
Case7	T/T	T/T	G/G	G/G	G/G	T/T	G/G	G/G	G/G	C/C	C/C	G/G	A/A	1/1	C/C	C/C
Case8	T/T	T/T	A/G	T/T	A/G	T/T	G/G	G/G	G/G	C/C	C/C	A/G	A/G	A/G	G/G	C/T
Case9	C/C	T/T	A/A	G/T	G/G	C/T	G/G	G/G	G/G	C/C	C/C	A/A	A/G	A/G	G/G	C/C
Case10	C/C	T/T	A/A	T/T	G/G	T/T	G/G	G/G	G/G	C/C	C/C	A/A	A/A	1/1	C/G	C/C
Case11	C/C	T/T	G/G	G/G	G/G	T/T	G/G	G/G	G/G	C/C	C/C	A/A	A/A	G/G	2/2	1/1
Case13	C/C	T/T	G/G	G/G	G/G	T/T	G/G	G/G	G/G	C/C	C/C	A/A	A/A	G/G	1/1	1/1

Fig. S9 Founder effect in 12 patients with homozygous c.1112A>G variant. Homozygosity mapping in 12 patients with c.1112A>G homozygote showed a 0.64Mb common region containing the variant, and further SNP analysis revealed a shared haplotype in the 0.64Mb region, suggesting a founder effect in these patients.

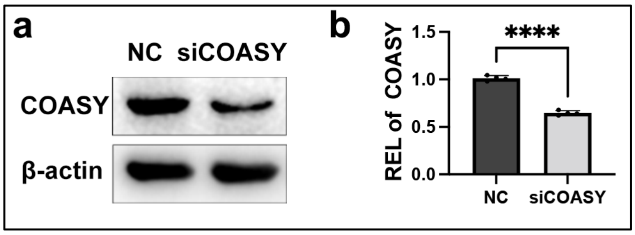

Fig. S10 *COASY* knockdown cell model. COASY-siRNA: 5'-CCUCUUGCAGAAGCGCAUUTT-3' significantly down-regulated COASY protein level in HEK293T cells (mean ± SD, n = 3, **** $p < 0.0001$).

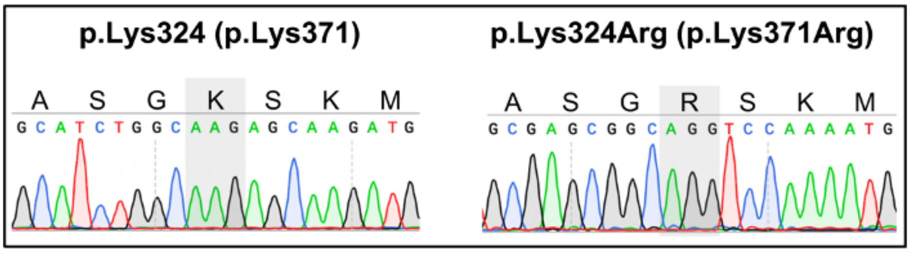

Fig. S11 The sequence of Ppat-Dpck in the knock-in *Drosophila*. Wild-type sequence of *Drosophila Ppat-Dpck* from W^{1118} (left), and variant sequence of *Ppat-Dpck* mutated by CRISPR/Cas9 system from W^{1118} (right), generating the p.Lys324Arg variant which is equivalent to p.Lys371Arg in patients with *COASY*-related RR-LSM.

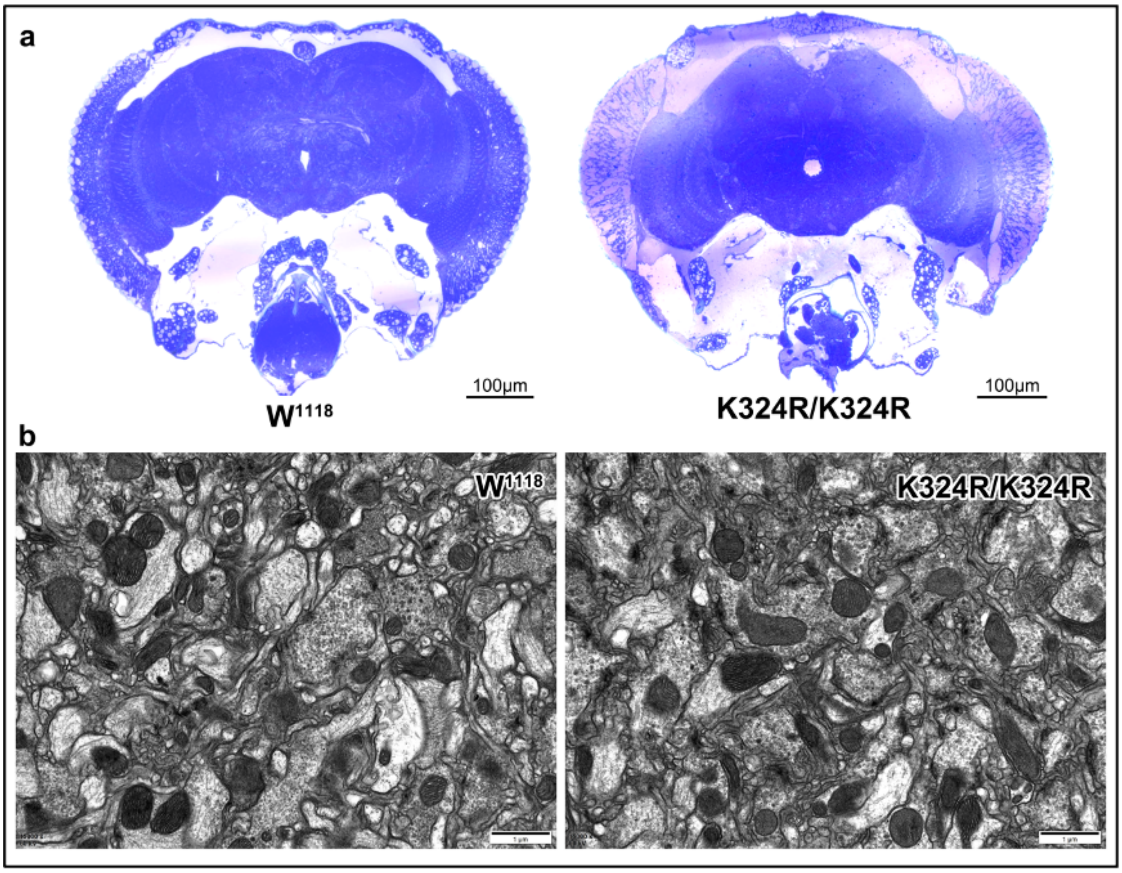

Fig. S12 Brain sections of fly models. **a** Semi-thin sections (1 μm thick) of a whole head revealed no detectable neurodegenerative vacuoles in the brain of K324R/K324R flies at 15-day-old. **b** Utral-thin sections showed no abnormalities on mitochondria in the brain of K324R/K324R flies.

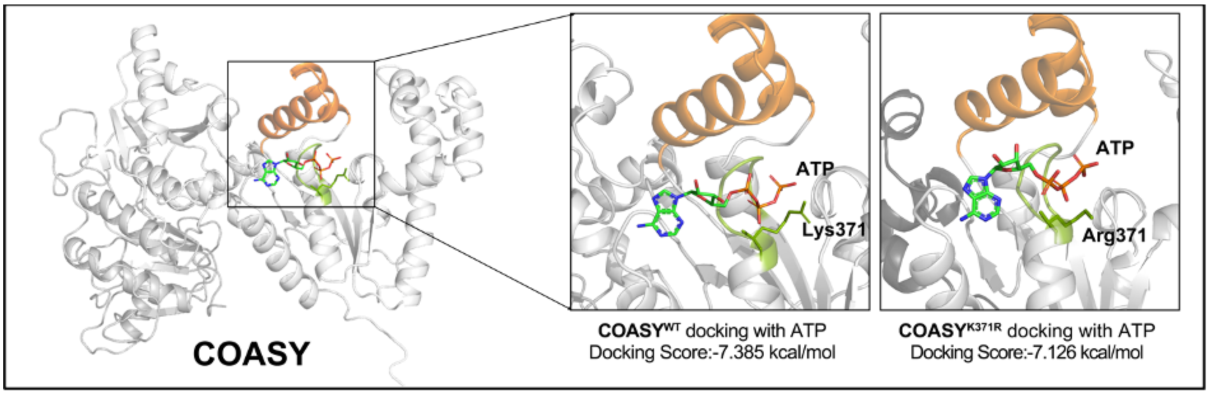

Fig. S13 The molecular docking result of human DPCK domain with ATP. The molecular docking of wild-type human DPCK domain with ATP (left). The variant of p.Arg371 might affect the hydrolysis of ATP in human DPCK (right).

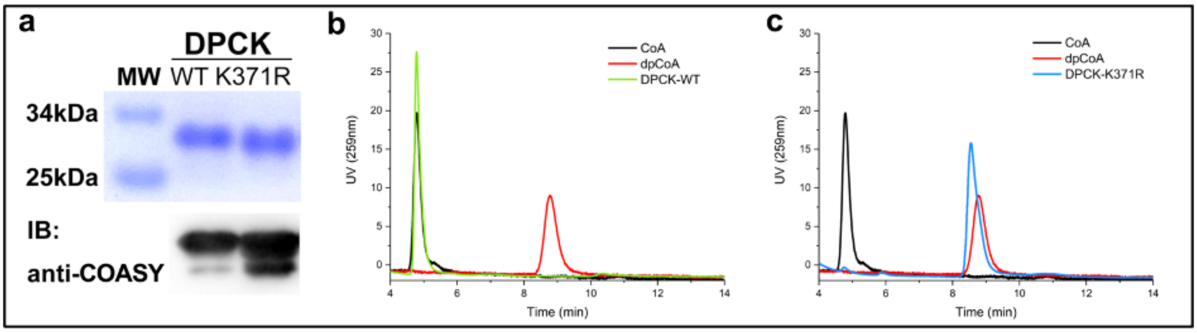

Fig. S14 Catalytic activity analysis of recombinantly human DPCK *in vitro*. a Equal amount of purified human DPCK^{WT} and DPCK^{K371R} proteins were loaded on SDS-PAGE and stained with Coomassie blue. Immunoblotting analysis on SDS-PAGE gel showed that anti-COASY antibody was able to recognize both DPCK^{WT} and DPCK^{K371R} proteins. **b** Chromatogram showed the standard peaks of CoA (black) and dephospho-CoA (red), as well as the peak corresponding to the reaction product of DPCK^{WT}. **c** Chromatograms showed the mutant hDPCK^{K371R} (blue) recombinant protein cannot convert dephospho-CoA to CoA.

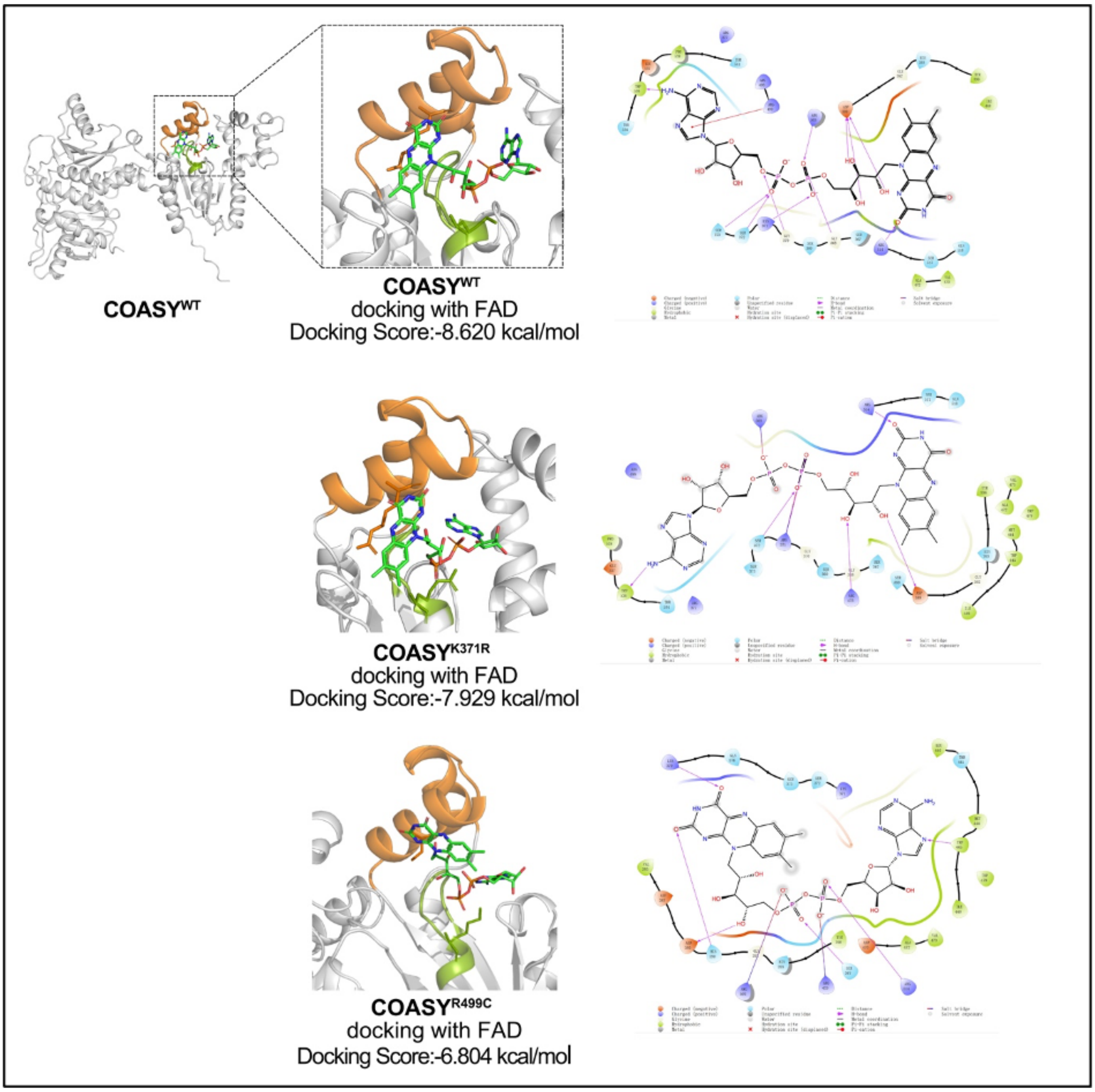

Fig. S15 MD studies of COASY^{WT}/COASY^{K371R}/COASY^{R499C} binding to FAD. Molecular docking scores indicated that FAD could effectively bind to the catalysis pocket of wild-type COASY and p.Lys371Arg mutant, while the p.Arg499Cys located in the LID region disrupted the conformation of substrate-binding pocket and blocked the binding of FAD.

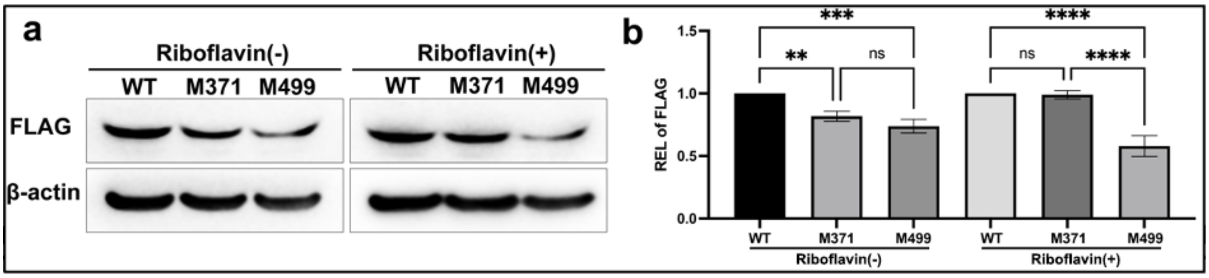

Fig. S16 Riboflavin for the stability of p.Lys371Arg or p.Arg499Cys mutant proteins. a,b In the cell models transfected with FLAG-tag full-length wild-type and COASY variant plasmids, p.Lys371Arg (M371) or p.Arg499Cys (M499) mutant protein levels significantly decreased, while the p.K371R mutant was significantly up-regulated after supplementing with riboflavin, but no detectable effects in p.Arg499Cys mutant.

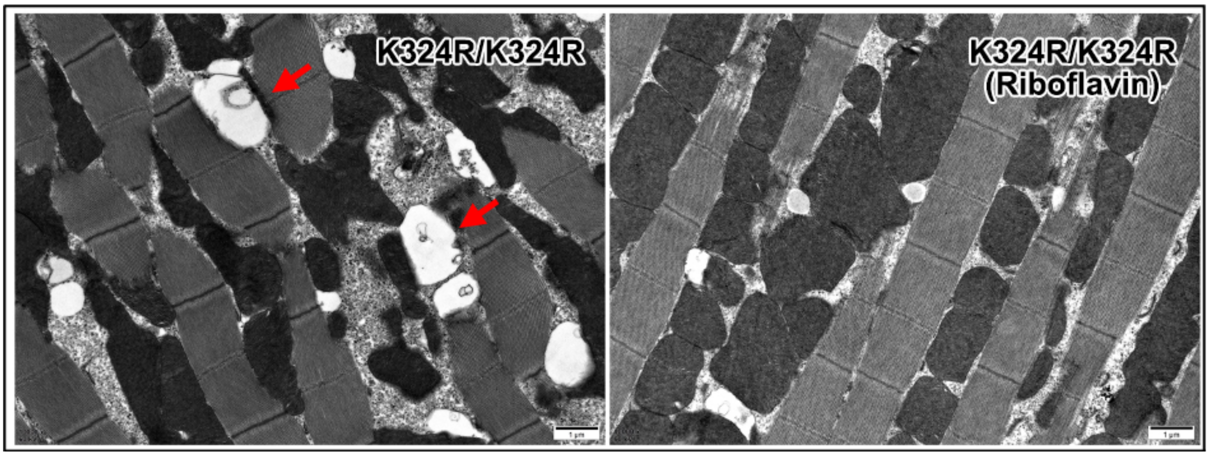

Fig. S17 The impact of riboflavin supplementation for *Drosophila*. In *Ppat-Dpck*^{K324R} knock-in flies, supplementation with 50 μM riboflavin resulted in substantial reduction in the accumulation of lipid droplets in muscles at day 20. LDs were indicated by red arrows.

Table S1 The demographic, clinical and laboratory data in 16 patients with biallelic *COASY* variants.

Case	Sex /age (year)	Disease duration	Trigger factors	Presenting symptom	Muscle weakness						Other symptoms	CK IU/L	Hcy μmol/L	EMG	COASY variant
					Masseter muscle	Neck	Proximal UL	Distal UL	Proximal LL	Distal LL					
1	F/20	6 months	Dieting	Muscle fatigue	4	4	4	5-	3	4	Myalgia	1423	27	Myogenic	c.1112A>G (homo)
2	M/22	1 month	High-fat diet	Muscle fatigue	5	5-	5-	5	4	5	CB, LN, PP	712	20	Normal	c.1112A>G (homo)
3	M/43	18 years	Cold	Leg weakness	4	4	5-	5-	4-	5-	VSD, FL, CM	1011	74	Myogenic	c.1112A>G (homo)
4	M/30	1 month	Diarrhea	Leg weakness	5	5	5	5	3	4	None	450	NA	Myogenic	c.1112A>G (homo)
5	F/32	8 months	None	Muscle fatigue	4	4	4	5	4	5-	Myalgia	932	NA	Normal	c.1112A>G (homo)
6	M/53	8 months	None	Muscle fatigue	5	5	5	5	4	5	Myalgia	8796	23.5	Myogenic	c.1112A>G (homo)
7	M/19	4 years	None	Muscle fatigue	3	3	3	5	3-	4+	Myalgia, Amyotrophy	2267	NA	Myogenic	c.1112A>G (homo)
8	M/56	4 years	None	Muscle fatigue	NA	5	5	5	5	5	Myalgia	5004	NA	Myogenic	c.1112A>G (homo)
9	M/29	3 years	None	Muscle fatigue	NA	5	5	5	5	5	Myalgia, CM	1343	21	Myogenic	c.1112A>G (homo)
10	F/29	5 years	None	Muscle fatigue	NA	5	4	4	3-	3-	Dysphagia	1772	25	Myogenic	c.1112A>G (homo)
11	M/21	2 years	None	Muscle fatigue	5	4	4	5	4	5	Soreness	625	NA	Myogenic	c.1112A>G (homo)
12	F/19	3 years	None	Muscle fatigue	5	5	5	5	4	5	PP	724	15.3	Normal	c.1112A>G (homo)
13	M/17	2 years	Vomiting	Muscle fatigue	5	5	5	5	4	5-	Fasciculation	61	15.6	Normal	c.1112A>G (homo)
14	M/20	3 months	Heavy work	Muscle fatigue	5	4	4	5	3	4	Myalgia	1365	NA	Myogenic	c.1112A>G; c.805_806insC
15	F/19	2 years	None	Leg weakness	NA	4	4	5	4	4	NA	260	NA	Myogenic	c.1112A>G; c.1018_1019insA
16	F/38	13 years	None	Muscle fatigue	NA	5	4	5	3	4	NA	560	NA	Myogenic	c.1112A>G; c.383C>T

Abbreviations: CB: compulsive behaviour; CK, creatine kinase; CM: cardiomyopathy; EMG, electromyogram; F, female; FL: fatty liver; Hcy: homocysteine; homo: homozygous; LL, lower limb; LN: limb numbness; M, male; NA, not available; PP: palpitation; UL, up limbs; VL, Visual loss; VSD: vibration sensation decrease.

Muscle strength was assessed according to the modified Medical Research Council scale.

Table S2 Plasma acylcarnitine and urine organic acid profiles in patients with *COASY* variants.

Case.	Amino acid	Acylcarnitine	Organic acids*
1	Glu↑ Gln↓	C0↑<br>C10:1, C16, C16:2, C18:1↓	Oxalic acid, glyceric acid, PAA, 3HG, PA
2	Ser↑ Gln↓	C10:1 ↓	Glycolic acid, oxalic acid, glyceric acid, 2M3HB, 3HG, 3HP, PA
2[a]	Glu, Ser↑ Gln↓	Normal	Glycolic acid, oxalic acid, 2HIB, 2HIV, 3HP, benzoin, glyceric acid, 3HG, PA
3	Glu, Gly↑ Gln↓	Normal	Oxalic acid, glyceric acid, PA, 2HG, 3HG
4	Arg, Cit, Met, Pro↓	C0↓<br>C8, C10, C12, C14, C14:1↑	3HG, 3HPA
5	NA	C5, C8, C12, C14:1, C14:2↑	Glutarate, adipate, suberate, 2HG
6	NA	C14, C16，C18:1OH，C14:1/C8:1↑	2HIV,4HPL
7	NA	C8, C10, C12 ↑	Glutarate, 2HIC, adipate, 2HG, 2HB, 2HIV, 2M3HB, 4HPL

[a] After treatment with riboflavin and coQ10; * more than reference level

2HB, 2-hydroxybutyrate; 2HG, 2-hydroxyglutaric acid; 2HIB, 2-Hydroxyisobutyric acid; 2HIC, 2-hydroxyhexanoic acid; 2HIV, 2-hydroxyisovaleric; 2M3HB, 2-methyl-3-hydroxybutyrate; 3HG, 3-hydroxyglutaric acid; 3HP, 3-hydroxypropanoic acid; 3HPA, 3-hydroxyphenylacetic acid; 3MGA, 3-methylglutaconic acid; 4HPL, 4-hydroxyphenylactic acid; PAA, phenylacetic acid; PA, palmitic acid.

Table S3 The clinical and genetic summarization in reported patients with *COSAY* variants.

Patient	Sex/Onset age	Recent Age	Clinical features					Other features	MRI features	Variant 1	Variant 2	Diagnosis	Ref
			MD	DD	CI	PS	AN						
1	F/2Y	25Y	++	+	++	+	+	Progressive dystonia, cognitive impairment, ambulate disorder	bilateral hypointensity in the globipallidi associated with a central region of hyperintensity in the anteromedial portion (“eye-of-the-tiger-sign")	c.1495C>T p.Arg499Cys	c.1495C>T p.Arg499Cys	CoPAN	Dusi, 2014[10]
2	M/1Y	19Y	+++	++	+++	+	+	gait difficulties, cognitive impairment, dysarthria and oro-mandibular dystonia	Bilateral hyperintensity and swelling of caudate nucleus, putamen; hypointensity in the globipallidi.	c.175C>T p.Gln59*	c.1495C>T p.Arg499Cys	CoPAN	Dusi, 2014[10]
3	F/4	17Y	+++	N/A	+++	+	+	gait difficulties, cognitive impairment, dysarthria and oro-mandibular dystonia	T2-hypointensity with central hyperintensity of GP (“eye-of-the-tiger-sign")	c.1495C>T p.Arg499Cys	c.1495C>T p.Arg499Cys	CoPAN	Annesi, 2016[13]
4	F/Infancy	8Y	++	+++	++	++	NA	hypotonia, broad based, ataxic gait, and ataxic hand movements, developmental delay, obsessive-compulsive symptoms and self-injurious behavior	Bilateral hyperintensity and swelling of caudate nucleus, putamen, and thalamus; small corpus callosum; Corpuscallosum was small and frontotemporal and parietal white matter changes	c.641 C>T p.Arg214Val	c.1495C>T p.Arg499Cys	CoPAN	Evers, 2017[14]
5	M/Infancy	7Y	++	+++	++	+	NA	hypotonia of the trunk, hyperreflexia, clonus of the Achilles tendon, and severe global developmental delay. Mild microcephaly.	bilateral T2- and FLAIR hyperintensity of nucleus caudatus, putamen, thalamus, and cortex with corresponding reduced ADC map	c.C641 C>T p.Arg214Val	c.1495C>T p.Arg499Cys	CoPAN	Evers, 2017[14]
6	NA	NA	NA	NA	NA	NA	NA	developmental delay/intellectual disability	NA	c.112dupT p.Tyr38Leufs*26	c.1495C>T p.Arg499Cys	CoPAN	Hiraide,T, 2021[15]
7	M/20W	Ter	NA	NA	NA	NA	NA	pontocerebellar hypoplasia, microcephaly, arthrogryposis	NA	c.1549_1550delA G p.Ser517Profs*61	c.1486-3C>G p.Ala496Ilefs*20	PCH	van Dijk, 2018[16]
8	M/28W	1M	NA	NA	NA	NA	NA	pontocerebellar hypoplasia, microcephaly, arthrogryposis, jittery	hypoplasia of cerebellum, brainstem and spinal cord,	c.1486-3C>G p.Ala496Ilefs*20	c.1486-3C>G p.Ala496Ilefs*20	PCH	van Dijk, 2018[16]
9	M/22W	Ter	NA	NA	NA	NA	NA	pontocerebellar hypoplasia, arthrogryposis	small cerebellum, brainstem, spinal cord, and basal ganglia	c.1486-3C>G p.Ala496Ilefs*20	c.1486-3 C > G p.Ala496Ilefs*20	PCH	van Dijk, 2018[16]

10	M/NA	Ter	NA	NA	NA	NA	NA	pontocerebellar hypoplasia,	NA	c.1486-3C>G p.Ala496Ilefs*20	c.1486-3 C > G p.Ala496Ilefs*20	PCH	van Dijk, 2018[16]
11	F/Infancy	NA (More then 20 months)	NA	NA	NA	NA	NA	Hypotonia, absent neonatal reflexes, arthrogryposis severely neurodevelopmentally delayed	loss of normal T1 hyperintensity and T2 hypointensity and diffusion abnormalities of the deep brain structure progressive diffuse parenchymal loss throughout the bilateral cerebral hemispheres and atrophy of the basal ganglia and brainstem	c.1664G>A p.Arg555His	c.1664G>A p.Arg555His	PCH	Rosati, J, 2023[17]
12	F/Infancy	NA (More then 94 days)	NA	NA	NA	NA	NA	Respiratory weakness，Hypotonia，severely neurodevelopmentally delayed	loss of normal T1 hyperintensity and T2 hypointensity and diffusion abnormalities of the deep brain structure	c.1664G>A p.Arg555His	c.1664G>A p.Arg555His	PCH	Rosati, J, 2023[17]

M, Male; F, Female; Y, Years; M, Month; W, Week; NA, not available; MD, movement disorders; DD, developmental delay; CI, cognitive impairment; PS, psychiatric symptoms; CoPAN, COASY protein-associated neurodegeneration; PCH, Pontocerebellar hypoplasia.

eye-of-the-tiger sign: Hypointensity in the globus pallidus bilaterally and hyperintensity in the anteromedial central region of the nucleus

+++, severe; ++ middle; +, mild; -, negative.

References

1. Jiang, K., Zheng, Y., Lin, J., Wu, X., Yu, Y., Zhu, M., Fang, X., Zhou, M., Li, X., and Hong, D. (2022). Diverse myopathological features in the congenital myasthenia syndrome with GFPT1 mutation. Brain Behav *12*, e2469.
2. Olsen, R.K., Olpin, S.E., Andresen, B.S., Miedzybrodzka, Z.H., Pourfarzam, M., Merinero, B., Frerman, F.E., Beresford, M.W., Dean, J.C., Cornelius, N., et al. (2007). ETFDH mutations as a major cause of riboflavin-responsive multiple acyl-CoA dehydrogenation deficiency. Brain *130*, 2045-2054.
3. Olsen, R.K.J., Konarikova, E., Giancaspero, T.A., Mosegaard, S., Boczonadi, V., Matakovic, L., Veauville-Merllie, A., Terrile, C., Schwarzmayr, T., Haack, T.B., et al. (2016). Riboflavin-Responsive and -Non-responsive Mutations in FAD Synthase Cause Multiple Acyl-CoA Dehydrogenase and Combined Respiratory-Chain Deficiency. Am J Hum Genet *98*, 1130-1145.
4. Schiff, M., Veauville-Merllié, A., Su, C.H., Tzagoloff, A., Rak, M., Ogier de Baulny, H., Boutron, A., Smedts-Walters, H., Romero, N.B., Rigal, O., et al. (2016). SLC25A32 Mutations and Riboflavin-Responsive Exercise Intolerance. N Engl J Med *374*, 795-797..
5. Quinodoz, M., Peter, V.G., Bedoni, N., Royer Bertrand, B., Cisarova, K., Salmaninejad, A., Sepahi, N., Rodrigues, R., Piran, M., Mojarrad, M., et al. (2021). AutoMap is a high performance homozygosity mapping tool using next-generation sequencing data. Nature Communications *12*, 518.
6. Bahlo, M., and Bromhead, C.J. (2009). Generating linkage mapping files from Affymetrix SNP chip data. Bioinformatics *25*, 1961-1962.
7. Abecasis, G.R., Cherny, S.S., Cookson, W.O., and Cardon, L.R. (2002). Merlin--rapid analysis of dense genetic maps using sparse gene flow trees. Nat Genet *30*.
8. Abraham, M.J., Murtola, T., Schulz, R., Páll, S., Smith, J.C., Hess, B., and Lindahl, E. (2015). GROMACS: High performance molecular simulations through multi-level parallelism from laptops to supercomputers. SoftwareX *1-2*, 19-25.
9. Tunyasuvunakool, K., Adler, J., Wu, Z., Green, T., Zielinski, M., Žídek, A., Bridgland, A., Cowie, A., Meyer, C., Laydon, A., et al. (2021). Highly accurate protein structure prediction for the human proteome. Nature *596*, 590-596.
10. Dusi, S., Valletta, L., Haack, T.B., Tsuchiya, Y., Venco, P., Pasqualato, S., Goffrini, P., Tigano, M., Demchenko, N., Wieland, T., et al. (2014). Exome sequence reveals mutations in CoA synthase as a cause of neurodegeneration with brain iron accumulation. Am J Hum Genet *94*, 11-22.
11. Walia, G., Gajendar, K., and Surolia, A. (2011). Identification of critical residues of the mycobacterial dephosphocoenzyme a kinase by site-directed mutagenesis. PLoS One *6*, e15228.
12. Yu, J., Liufu, T., Zheng, Y., Xu, J., Meng, L., Zhang, W., Yuan, Y., Hong, D., Charlet-Berguerand, N., Wang, Z., and Deng, J. (2022). CGG repeat expansion in NOTCH2NLC causes mitochondrial dysfunction and progressive neurodegeneration in Drosophila model. Proc Natl Acad Sci U S A *119*, e2208649119.
13. Annesi, G., Gagliardi, M., Iannello, G., Quattrone, A., Iannello, G., and Quattrone, A. (2016). Mutational analysis of COASY in an Italian patient with NBIA. Parkinsonism Relat Disord *28*, 150-151.
14. Evers, C., Seitz, A., Assmann, B., Opladen, T., Karch, S., Hinderhofer, K., Granzow, M., Paramasivam, N., Eils, R., Diessl, N., et al. (2017). Diagnosis of CoPAN by whole exome sequencing: Waking up a sleeping tiger's eye. Am J Med Genet A *173*, 1878-1886.
15. Hiraide, T., Yamoto, K., Masunaga, Y., Asahina, M., Endoh, Y., Ohkubo, Y., Matsubayashi, T., Tsurui, S., Yamada, H., Yanagi, K., et al. (2021). Genetic and phenotypic analysis of 101 patients with developmental delay or intellectual disability using whole-exome sequencing. Clin Genet *100*, 40-50.
16. van Dijk, T., Ferdinandusse, S., Ruiter, J.P.N., Alders, M., Mathijssen, I.B., Parboosingh, J.S., Innes, A.M., Meijers-Heijboer, H., Poll-The, B.T., Bernier, F.P., et al. (2018). Biallelic loss of function variants in COASY cause prenatal onset pontocerebellar hypoplasia, microcephaly, and arthrogryposis. Eur J Hum Genet *26*, 1752-1758.
17. Rosati, J., Johnson, J., Stander, Z., White, A., Tortorelli, S., Bailey, D., Fong, C.T., and Lee, B.H. (2023).

Progressive brain atrophy and severe neurodevelopmental phenotype in siblings with biallelic COASY variants. Am J Med Genet A *191*, 842-845.
